# Prospective functional assessment after mandible reconstruction and bone healing assessment

**DOI:** 10.1007/s00784-025-06698-3

**Published:** 2025-12-18

**Authors:** Philipp Ruf, Katharina Duda, Jakob Fenske, Vincenzo Orassi, Philipp Lampert, Henri Kreiker, Sara Checa, Max Heiland, Carsten Rendenbach, Claudius Steffen

**Affiliations:** 1 Department of Oral and Maxillofacial Surgery, Charité - Universitätsmedizin, corporate member of Freie Universität Berlin and Humboldt-Universität zu Berlin, Augustenburger Platz 1, Berlin, 13353 Germany; 2 Julius Wolff Institute, Berlin Institute of Health at Charité - Universitätsmedizin Berlin, Augustenburger Platz 1, Berlin, 13353 Germany; 3 Institute of Biomechanics, Hamburg University of Technology (TUHH), Denickestrasse 15, Hamburg, 21073 Germany

**Keywords:** Mandibular reconstruction, Functional analysis, Bite force, Biomechanics, Bone healing

## Abstract

**Objectives:**

Mandibular reconstruction remains a clinical challenge due to a high rate of osseous non-unions. Mechanical factors are known to influence the bone regeneration process. Therefore, bite force levels, both immediately after surgery and during the bone healing process, are believed to play a critical role in the healing outcome. The aim of this study was to evaluate bite force and mandibular mobility measurements over time following mandibular reconstruction and to relate these measurements to the bone healing outcome.

**Materials and methods:**

In this prospective study, 28 patients were included and underwent functional measurements pre-operatively and at two weeks, four weeks, eight weeks, three months, and six months post-reconstruction. Additionally, the healing outcome was assessed using post-operative imaging.

**Results:**

After surgery, the functional parameters - including bite force, mouth opening, protrusion, and laterotrusion - significantly decreased. However, between the initial post-operative measurement and the final follow-up six months after surgery, all parameters showed a significant increase. Two weeks post-operatively, only 9 of the 28 patients were able to participate in the bite force measurement. However, 6 of these patients exhibited incomplete osseous union, which was associated with a mean bite force of 166 N at two weeks (mean over the whole cohort after six months of 154 N).

**Conclusions:**

This study demonstrated the trends in functional measurements over time following mandibular reconstruction and identified a significant trend in association between excessive post-operative bite force and incomplete osseous union.

**Clinical relevance:**

In consequence, clinical strategies to reduce mechanical over-stimulation could be evaluated in future studies, such as soft food intake for 3 months after mandibular reconstruction.

## Introduction

Mandibular reconstruction due to tumor resection, osteoradionecrosis or medication induced osteonecrosis represents a clinical challenge [1]. In particular, the development of osseous non-union after surgery can prolong the clinical course [2–4]. When the mandible and the vascularized bone graft do not heal sufficiently, plate removal is not possible, which consequently compromises the placement of dental implants and, therefore, dental rehabilitation [5]. Biomechanical factors are known to be a major driver of bone regeneration [6–9]. In consequence, the effect of biomechanical factors on osseous non-union is a major subject in the field of mandibular reconstruction [3, 4, 10–12]. Previous studies evaluated the biomechanical conditions in the healing reconstructed mandible using animal models, biomechanical testing or computational approaches [13–16]. Most of these studies rely on long-term bite force measurements as an input factor [17–19], since, to our knowledge, no bite force evaluation in the initial healing phase after mandibular reconstruction has been performed before [12]. Paradoxically, the mechanical conditions in the initial healing phase are known to be a critical determinant of the bone healing outcome [8, 9]. Furthermore, to our knowledge, the influence of bite force - which has been described as highly variable in patients undergoing mandibular reconstruction [17, 18, 20] - on bone healing has not yet been investigated. Using a novel, previously validated device for bite force measurement, it has recently been demonstrated that bite force measurement during the clinical course after mandibular reconstruction is feasible [20].

Therefore, the present study aimed to prospectively assess bite force along with mandibular mobility before the reconstructive surgery and in the clinical course (primary end point). Furthermore, bone healing in the patients was assessed as a secondary end point in the clinical course to relate the functional measurements to the clinical outcomes.

## Methods

**Fig. 1:**
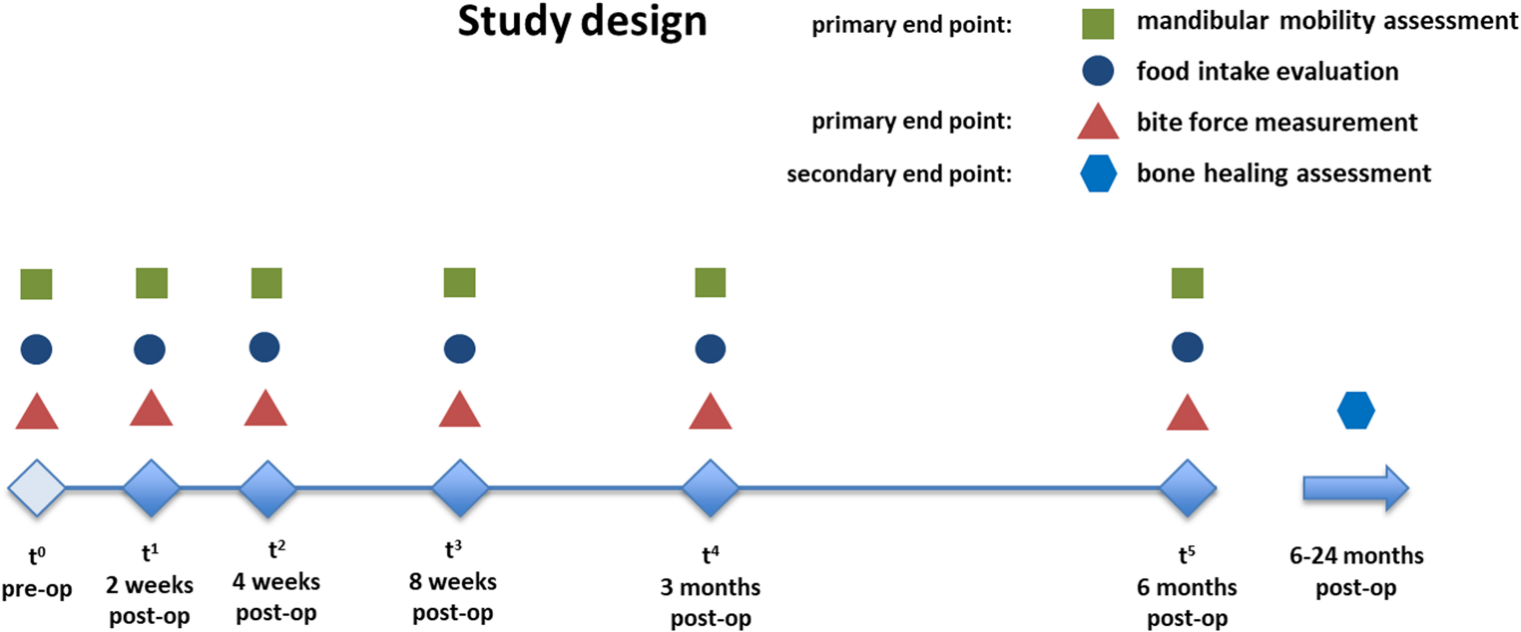
Graphical description of the study design including the primary end points of the functional measurements, the secondary end point of bone healing assessment and the food intake evaluation

The study design is graphically described in Fig. 1.

### Patients

In the present prospective study, 28 patients who underwent mandibular reconstruction using vascularized bone grafts at the department of oral and maxillofacial surgery at Charité - Universitätsmedizin Berlin between July 2022 and March 2023 were included. An ethics approval was received at Charité – Universitätsmedizin Berlin (EA2/138/18). After obtaining informed consent, patients were monitored over a six-month period, with the initial examination taking place pre-operatively (t^0^). Additional examination points were set at two weeks (t^1^), four weeks (t^2^), eight weeks (t^3^), three months (t^4^), and six months (t^5^) post-reconstruction (Fig. 1).

Exclusion criteria included age below 18 years, incapability to give informed consent, insufficient data due to early flap loss, non-compliance for participation in a prospective study, voluntary withdrawal, or death within the first six months post-surgery.

Questionnaires were filled out by the patients to obtain the data regarding nicotine and alcohol abuse. Clinical patient data such as surgery dates, diagnosis and information on adjuvant radio- or chemotherapy was collected from the internal clinical database (SAP Deutschland SE & Co. KG, Walldorf, Germany). Furthermore, the dental status was pre-operatively examined.

Digital planning documents from KLS Martin (KLS Martin SE & Co. KG, Tuttlingen, Germany) and DePuy Synthes (Johnson & Johnson Medical GmbH, Norderstedt, Germany) were evaluated in order to obtain data on the extent of the resection, number of segments as well as type and number of plates and screws.

### Mandibular mobility assessment

The clinical examination assessed mandibular movements in the sagittal, transverse, and vertical planes. Maximum protrusion and laterotrusion were measured using a steel ruler relative to the habitual occlusion. Maximum mouth opening was determined by measuring the incisal distance in dentate patients, while in edentulous patients, the distance between the upper and lower alveolar ridges was measured.

### Bite force measurement

A prototype for bite force measurement was previously developed and validated [20]. To prevent sensor damage, silicone bite pads were customized for each patient, thus achieving equal measurement conditions for dentulous and edentulous patients. The bite force device was placed between these silicone inserts, and patients were instructed to bite down with maximum force three times. The maximum bite force of the three measurements was chosen for analysis. Additionally, qualitative data regarding food intake was collected for each time point using a questionnaire with the options normal and soft food intake along with the option of invasive feeding.

### Bone healing assessment

The assessment of post-operative ossification as a secondary end point with observational character was performed based on radiographs in the clinical course (CT/CBCT/OPT) [21]. One investigator screened for incomplete osseous union (JF), blinded to functional measurements. Data was acquired for two time points: the first between 6 and 12 months after surgery and the second between 12 and 24 months after surgery. Analogously to a previous study investigating ossification outcomes, the term “incomplete osseous union” has been defined as follows: “≥1 intersegmental gap with less than 50% radiographic ossification at least 6 months after surgery (diagnosed in CBCT, CT or OPT scans with decreasing priority)” [21].

### Data analysis

Patient data, along with data from functional analyses, questionnaires, planning documents and the clinical database were imported into SPSS (IBM, Armonk, United States) for formal analysis. Missing values were excluded for each analysis (pair-wise for dependent analyses). For all statistical analyses, a two-sided significance threshold of *p* < 0.05 was employed. Graphical data presentation has been conducted using GraphPad Prism 10 (GraphPad Software, Boston, United States). For continuous variables, normal distribution was assessed using the Shapiro-Wilk test. To account for missing normal distribution while preserving comparability, Wilcoxon signed-rank tests were applied to compare functional measurements over time. Mann-Whitney-U tests were performed to compare continuous variables between groups. For group comparison involving categorial variables, Chi-squared tests were used. When at least one expected value was below 5, Fisher’s exact test was applied instead. Observational multivariate regression analysis was conducted using binary logistic regression to evaluate the influence of factors - either previously reported or identified as significant in univariate analyses - on the development of incomplete osseous union between 6 and 12 months post-operatively. Odds ratios (OR), 95% confidence intervals (95%-CI), and p-values were reported for each predictor. Additionally, a receiver operating characteristic (ROC) curve was generated, and the corresponding area under the curve (AUC) was calculated to assess the predictive value of the regression model. No multivariate regression was performed for incomplete osseous union between 12 and 24 months due to the low number of positive events.

## Results

### Cohort characteristics

The cohort characteristics are demonstrated in Table 1. A total of 33 patients were initially enrolled and examined pre-operatively. Five patients withdrew from or were excluded from the study according to the exclusion criteria. 18 included subjects were men and 10 subjects were women with a mean age of 63 years. The most common donor site was the fibula and the most common defect types according to the HCL classification were L and LC [22]. Plating systems used for fixation were singular patient-specific titanium reconstruction plates or a combination of posterior patient-specific titanium reconstruction plates and anterior patient-specific titanium miniplates (Mix) [23]. Most patients were not fully dentulous pre-operatively and the most common indication for surgery was oral squamous cell carcinoma. 13 patients underwent post-operative radiotherapy. Two patients received a post-operative chemotherapy additional to the radiotherapy while one patient received a peri-operative chemotherapy without any radiotherapy. A history of nicotine or alcohol abuse was reported by many patients.

**Table 1:** Cohort characteristics with frequencies or mean and standard deviation (SD)

Variables (*N*)	Characteristics	Frequencies/Mean +- SD
Sex (*N* = 28)	Female	10 (35.7%)
	Male	18 (64.3%)
Age in years (*N* = 28)	Mean ± SD	62.9 ± 13
Previous head and neck surgery (*N* = 28)	No	23 (82.1%)
	Yes	5 (17.9%)
Flap type (*N* = 28)	Fibula	26 (92.8%)
	Scapula	1 (3.6%)
	Iliac crest	1 (3.6%)
HCL-Classification (*N* = 28)	L	8 (28.6%)
	H	1 (3.6%)
	LC	8 (28.6%)
	HC	5 (17.8%)
	LCL	6 (21.4%)
Number of segments (*N* = 28)	One segment	5 (17.9%)
	Two segments	9 (32.1%)
	Three segments	14 (50%)
Total flap length in mm (*N* = 28)	Mean ± SD	103.6 ± 28.3
Post-operative dental status (*N* = 28)	Edentulous	8 (28.6%)
	Partial denture without contact zones	8 (28.6%)
	Parital denture with 1–2 contact zones	11 (39.2%)
	At least 3 contact zones	1 (3.6%)
Reason for resection (*N* = 28)	Squamous cell carcinoma	20 (71.4%)
	Osteoradionecrosis	2 (7.1%)
	Osteomyelitis	3 (10.7%)
	Osteosarcoma	1 (3.6%)
	Ameloblastoma	1 (3.6%)
	Chondrosarcoma	1 (3.6%)
Plate system (*N* = 28)	Posterior reconstruction plate with anterior miniplates (Mix)	23 (82.1%)
	Reconstruction plate	5 (17.9%)
Number of miniplates (*N* = 28)	None	5 (17.9%)
	Two miniplates	7 (25%)
	Four miniplates	16 (57.1%)
Number of reconstruction plates (*N* = 28)	One reconstruction plate	24 (85.7%)
	Two reconstruction plates	4 (14.3%)
Total number of screws (*N* = 28)	Mean ± SD	20.6 ± 5.7
Post-operative radiotherapy (*N* = 28)	No	15 (53.6%)
	Yes	13 (46.4%)
Nicotine abuse (*N* = 28)	No	9 (32.1%)
	Terminated nicotine abuse	9 (32.1%)
	Recent nicotine abuse	10 (35.8%)
Alcohol abuse (*N* = 28)	No	8 (28.6%)
	Terminated alcohol abuse	4 (14.3%)
	Recent alcohol abuse	16 (57.1%)
Incomplete osseous union 6–12 months (*N* = 24)	No	14 (58.3%)
	Yes	10 (41.7%)
Incomplete osseous union 12–24 months (*N* = 23)	No	20 (87%)
	Yes	3 (13%)

#### Functional measurements over time

**Fig. 2:**
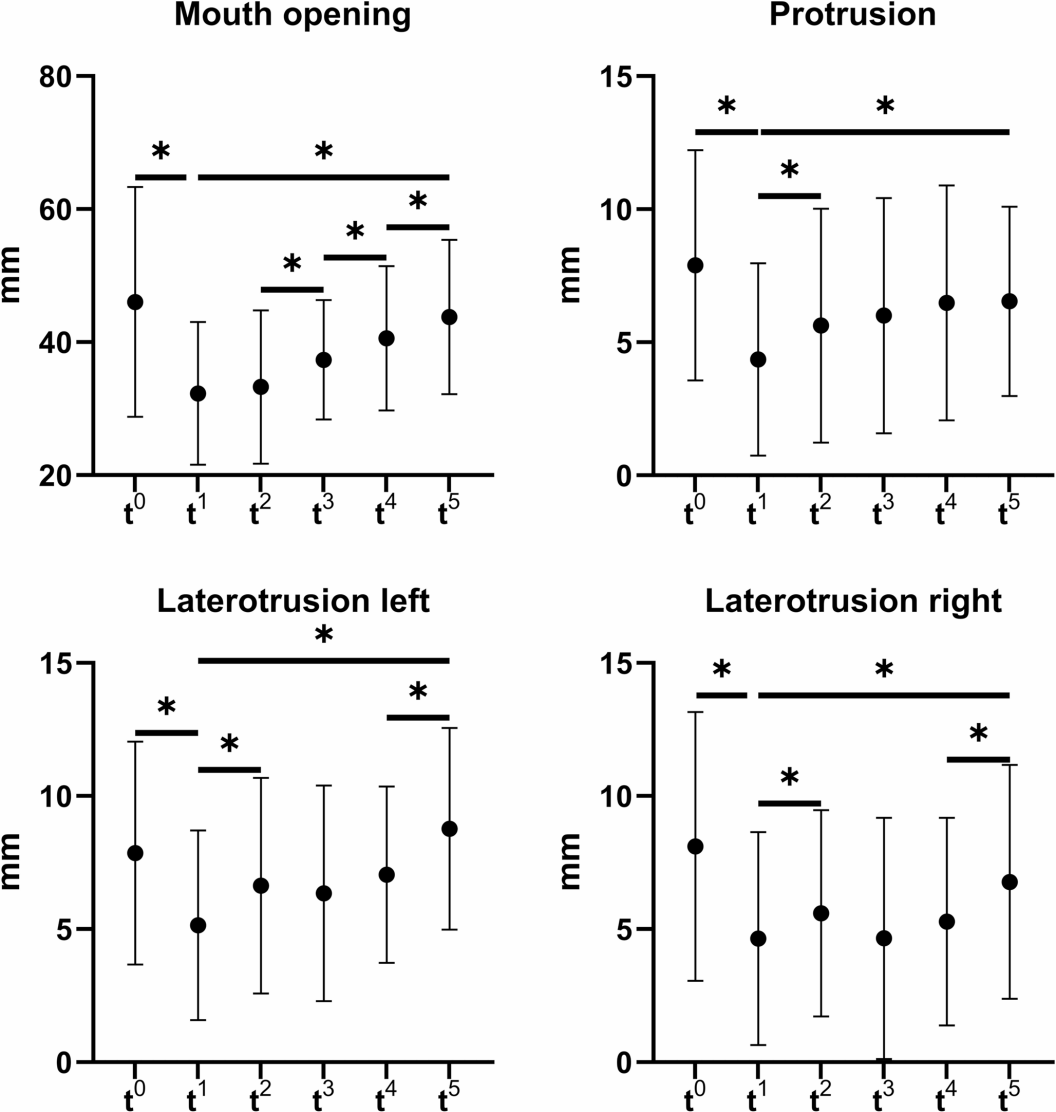
Development of mandibular movement parameters over time (points: mean, ranges: standard deviations) for the time points t^0^ (pre-operative), t^1^ (2 weeks after surgery), t^2^ (four weeks after surgery), t^3^ (eight weeks after surgery), t^4^ (three months after surgery), and t^5^ (six months post-reconstruction); significant differences marked for Wilcoxon-tests (*) on a significance level of *p* < 0.05

**Fig. 3:**
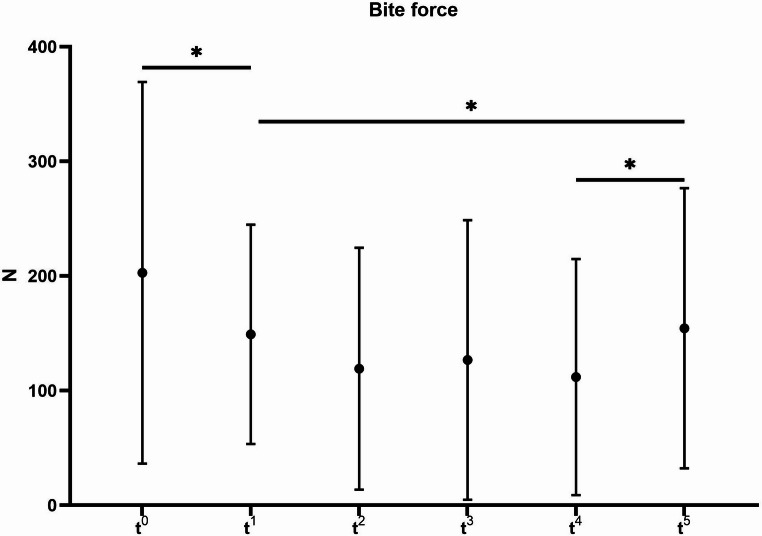
Development of bite force over time (points: mean, ranges: standard deviations) for the time points t^0^ (pre-operative), t^1^ (2 weeks after surgery), t^2^ (four weeks after surgery), t^3^ (eight weeks after surgery), t^4^ (three months after surgery), and t^5^ (six months post-reconstruction); significant differences marked for Wilcoxon-tests (*) on a significance level of *p* < 0.05

**Fig. 4:**
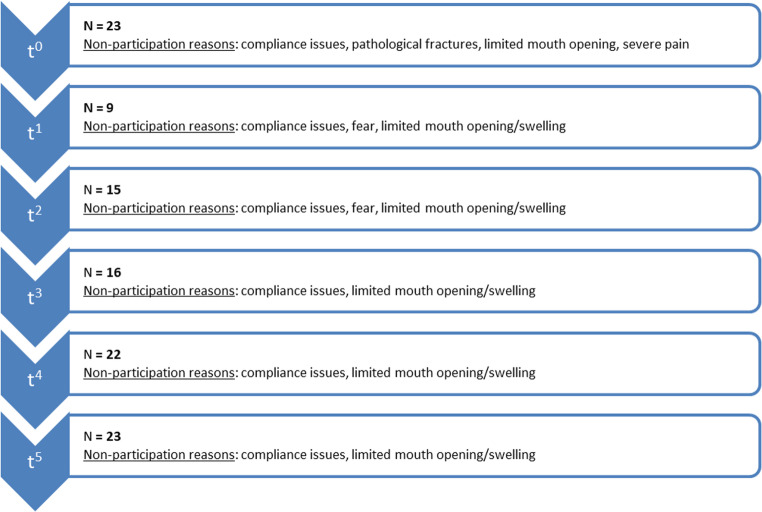
Reasons for non-participation in bite force measurement at the different time points t^0^ (pre-operative), t^1^ (2 weeks after surgery), t^2^ (four weeks after surgery), t^3^ (eight weeks after surgery), t^4^ (three months after surgery), and t^5^ (six months post-reconstruction)

The development of the functional measurements over time are presented in Fig. 2 for the movement parameters and in Fig. 3 for the bite force. As a result of the surgery, both the movement parameters as well as the bite force experienced a significant decrease. The p-values between the pre-operative measurement (t^0^) and the initial post-operative measurement 2 weeks after surgery (t^1^) were 0.015 for bite force, < 0.001 for mouth opening, protrusion and laterotrusion to the right and 0.004 for laterotrusion to the left. The mean bite force of 23 patients at t^0^ was 202.5 N whereas at t^1^ 9 patients were measured with a mean bite force of 148.8 N. The means of the bite force from t^2^ were 118.9 N (15 patients at t^2^), 126.5 N (16 patients at t^3^), 111.6 (22 patients at t^4^) and 154.1 N (23 patients at t^5^). The reasons for non-participation of patients at the different time points along with the participation rates are presented in Fig. 4. Between the initial measurement 2 weeks after surgery (t^1^) and the final measurement 6 months after surgery (t^5^) all functional parameters experienced a significant increase represented by p-values of 0.008 for bite force, < 0.001 for mouth opening and laterotrusion to the left, 0.003 for protrusion and 0.004 for laterotrusion to the right. No parameter differed significantly between the pre-operative (t^0^) measurement and the measurement 6 months after surgery (t^5^). All parameters except protrusion showed a significant increase between the measurements 3 months (t^4^) and 6 months (t^5^) after surgery (p-values of < 0.001 for bite force, mouth opening and laterotrusion to the left and 0.014 for laterotrusion to the right).

The relation of dentition and bite force is reported in Table 2. Edentulous patients had a significantly reduced bite force in comparison to patients with at least partial denture pre-operatively and in the functional measurements at 3 and 6 months post-operatively. Although not significant due to the lack of comparison, only patients with at least partial denture were able to participate in the initial bite force measurement.

**Table 2:** Univariate analysis of bite force in N at all time points grouped after dental status with p-values

	Dental status		
	Edentulous Mean ± SD (*N*)	At least partial dentition Mean ± SD (*N*)	*p*-value
Bite force t^0^ (*N* = 23)	82,1 ± 15,9 (*N* = 7)	255,2 ± 43,4 (*N* = 16)	0.012*
Bite force t^1^ (*N* = 9)	-	148,8 ± 31,9 (*N* = 9)	-
Bite force t^2^ (*N* = 15)	41 ± 14 (*N* = 3)	138,4 ± 31,6 (*N* = 12)	0.136
Bite force t^3^ (*N* = 16)	39,3 ± 11,9 (*N* = 4)	155,5 ± 37,1 (*N* = 12)	0.133
Bite force t^4^ (*N* = 22)	48,6 ± 6,2 (*N* = 6)	135,3 ± 28 (*N* = 16)	0.008*
Bite force t^5^ (*N* = 23)	70,1 ± 7,8 (*N* = 6)	183,8 ± 31,4 (*N* = 17)	0.01*

#### Influence of functional measurements on bone formation

The influence of the continuous parameters on bone formation was evaluated using univariate analyses (Table 3). Age had no significant association with incomplete osseous union in the present patient cohort. Significantly higher values for mouth opening during the clinical follow-up (from eight weeks post-surgery onward) were observed in the group with complete osseous union compared to the group with incomplete osseous union. For the three parameters - protrusion, laterotrusion to the left, and laterotrusion to the right - only isolated statistically significant differences in parameter magnitudes at specific time points between the osseous union groups were observed. In contrast, for bite force, patients with incomplete osseous union consistently demonstrated significantly higher bite force values. Among the patients who were able to bite immediately after surgery, six showed incomplete osseous union between six and twelve months, with a mean bite force of 165.8 N, while only one patient achieved complete osseous union with a bite force of 44 N (*p* = 0.286). Four weeks after surgery, the average bite force exceeded 200 N in the incomplete osseous union group (*N* = 7), compared to 42 N (*N* = 6) in the group with complete osseous union between six and twelve months (*p* = 0.001), indicating a strong trend towards association of excessive bite force and incomplete osseous union in the present patient collective.

**Table 3:** Univariate analysis of continuous parameters (in years, mm and N) at all time points grouped after occurrence of incomplete osseous union between 6 and 12 months and 12-24 months with p-values

	**Normal distribution** (Shapiro-Wilk)	**(Subtotal) osseous non-union 6-12 months** (N = 24)			**(Subtotal) osseous non-union 12-24 months** (N = 23)		
		**No** (N = 14)	**Yes** (N = 10)	**p-value**	**No **(N = 20)	**Yes **(N = 3)	**p-value**
		**Mean ± SD (N)**	**Mean ± SD (N)**		**Mean ± SD (N)**	**Mean ± SD (N)**	
Age (*N* = 28)	Yes	64.6 ± 13.8 (*N* = 14)	57.4 ± 12.6 (*N* = 10)	0.196	65 ± 11.9 (*N* = 20)	61.3 ± 10.2 (*N* = 3)	0.612
Mouth opening t^0^ (*N* = 28)	Yes	47.9 ± 21.3 (*N* = 14)	46.5 ± 11.1 (*N* = 10)	0.546	46.5 ± 18.1 (*N* = 20)	52.3 ± 11.7 (*N* = 3)	0.635
Mouth opening t^1^ (*N* = 28)	Yes	33.4 ± 12.9 (*N* = 14)	30.7 ± 78 (*N* = 10)	0.371	34 ± 10.9 (*N* = 20)	26 ± 3.6 (*N* = 3)	0.076
Mouth opening t^2^ (*N* = 27)	Yes	36.6 ± 10.6 (*N* = 14)	29.7 ± 12.7 (*N* = 10)	0.235	36.4 ± 9 (*N* = 20)	27 ± 6.1 (*N* = 3)	0.046*
Mouth opening t^3^ (*N* = 23)	Yes	40.9 ± 8.5 (*N* = 13)	31.9 ± 8.5 (*N* = 8)	0.016*	39.4 ± 7.9 (*N* = 17)	25.7 ± 5.1 (*N* = 3)	0.007*
Mouth opening t^4^ (*N* = 25)	Yes	45 ± 11.6 (*N* = 13)	35 ± 8.5 (*N* = 8)	0.013*	42.4 ± 10.7 (*N* = 18)	29.7 ± 5 (*N* = 3)	0.017*
Mouth opening t^5^ (*N* = 25)	Yes	49.7 ± 12.1 (*N* = 12)	38.1 ± 8.9 (*N* = 9)	0.018*	46.3 ± 11 (*N* = 18)	30.7 ± 6.8 (*N* = 3)	0.006*
Protrusion t^0^ (*N* = 28)	Yes	8.4 ± 4.5 (*N* = 14)	8.8 ± 3.8 (*N* = 10)	0.752	7.7 ± 4.3 (*N* = 20)	12 ± 3.5 (*N* = 3)	0.139
Protrusion t^1^ (*N* = 28)	No	5.1 ± 4.6 (*N* = 14)	3.9 ± 2.3 (*N* = 10)	0.841	4.8 ± 4 (*N* = 20)	4.3 ± 2.3 (*N* = 3)	0.83
Protrusion t^2^ (*N* = 27)	No	5.4 ± 4.7 (*N* = 14)	6.6 ± 4.5 (*N* = 10)	0.472	5.6 ± 4.4 (*N* = 20)	8.7 ± 6 (*N* = 3)	0.355
Protrusion t^3^ (*N* = 23)	No	6.1 ± 5.1 (*N* = 13)	6.6 ± 3.5 (*N* = 8)	0.645	6.1 ± 4.7 (*N* = 17)	7.7 ± 4 (*N* = 3)	0.546
Protrusion t^4^ (*N* = 25)	Yes	7.2 ± 5.1 (*N* = 13)	5.4 ± 3.5 (*N* = 8)	0.547	6.5 ± 4.9 (*N* = 18)	5.7 ± 2.3 (*N* = 3)	1
Protrusion t^5^ (*N* = 26)	Yes	7.2 ± 3.9 (*N* = 13)	5.6 ± 2.8 (*N* = 9)	0.324	6.4 ± 3.9 (*N* = 19)	6 ± 1 (*N* = 3)	0.929
Laterotrusion left t^0^ (*N* = 28)	Yes	8.1 ± 4.4 (*N* = 14)	9.1 ± 3.7 (*N* = 10)	0.585	7.6 ± 4.4 (*N* = 20)	12.7 ± 0.6 (*N* = 3)	0.076
Laterotrusion left t^1^ (*N* = 28)	Yes	4.1 ± 3.5 (*N* = 14)	6.3 ± 3.7 (*N* = 10)	0.138	4.7 ± 3.6 (*N* = 20)	8 ± 3.6 (*N* = 3)	0.166
Laterotrusion left t^2^ (*N* = 27)	Yes	5.6 ± 3.8 (*N* = 14)	9.1 ± 3.4 (*N* = 10)	0.031*	6.2 ± 4.2 (*N* = 20)	11 ± 2.6 (*N* = 3)	0.06*
Laterotrusion left t^3^ (*N* = 23)	Yes	5.5 ± 3.3 (*N* = 13)	8.6 ± 4.7 (*N* = 8)	0.14*	6.2 ± 4.2 (*N* = 17)	9.7 ± 3.1 (*N* = 3)	0.179
Laterotrusion left t^4^ (*N* = 25)	Yes	6.5 ± 3.4 (*N* = 13)	8.6 ± 2.8 (*N* = 8)	0.161	6.4 ± 3.3 (*N* = 18)	10.3 ± 3.2 (*N* = 3)	0.125
Laterotrusion left t^5^ (*N* = 26)	No	7.9 ± 2.6 (*N* = 13)	11 ± 4.4 (*N* = 9)	0.06*	8 ± 2.9 (*N* = 19)	13.33 ± 7.6 (*N* = 3)	0.226
Laterotrusion right t^0^ (*N* = 28)	Yes	7.9 ± 4.9 (*N* = 14)	9.5 ± 5.1 (*N* = 10)	0.472	7.5 ± 4.9 (*N* = 20)	15 ± 0 (*N* = 3)	0.012*
Laterotrusion right t^1^ (*N* = 28)	No	3.6 ± 3.7 (*N* = 14)	6 ± 4.2 (*N* = 10)	0.172	4.4 ± 4 (*N* = 20)	6.7 ± 5.5 (*N* = 3)	0.404
Laterotrusion right t^2^ (*N* = 27)	Yes	4.7 ± 4.3 (*N* = 14)	7.4 ± 2.7 (*N* = 10)	0.048*	5.2 ± 4.2 (*N* = 20)	8.3 ± 2.9 (*N* = 3)	0.23
Laterotrusion right t^3^ (*N* = 23)	No	3.2 ± 3 (*N* = 13)	7.9 ± 5.5 (*N* = 8)	0.037*	4.4 ± 4.8 (*N* = 17)	7 ± 5.2 (*N* = 3)	0.546
Laterotrusion right t^4^ (*N* = 25)	No	5.5 ± 4.4 (*N* = 13)	5.1 ± 3 (*N* = 8)	1	5.2 ± 4.1 (*N* = 18)	5.3 ± 2.9 (*N* = 3)	0.814
Laterotrusion right t^5^ (*N* = 26)	No	6 ± 3.6 (*N* = 13)	8.2 ± 5 (*N* = 9)	0.393	6.4 ± 4.1 (*N* = 19)	8.7 ± 6.5 (*N* = 3)	0.718
Bite force t^0^ (*N* = 23)	Yes	139.4 ± 86.2 (*N* = 11)	332.3 ± 189.8 (*N* = 8)	0.033*	188.9 ± 174.1 (*N* = 16)	268.2 ± 151.2 (*N* = 3)	0.359
Bite force t^1^ (*N* = 9)	No	44 ± 0 (*N* = 1)	165.8 ± 86.1 (*N* = 6)	0.286	145.8 ± 120.7 (*N* = 3)	156.8 ± 100.4 (*N* = 3)	1
Bite force t^2^ (*N* = 15)	No	42.3 ± 28 (*N* = 6)	206.5 ± 92.3 (*N* = 7)	0.001*	86 ± 85.2 (*N* = 10)	196.5 ± 116.2 (*N* = 3)	0.077
Bite force t^3^ (*N* = 16)	No	52.7 ± 39.1 (*N* = 9)	251.8 ± 109.9 (*N* = 6)	< 0.001*	89 ± 106 (*N* = 12)	188.5 ± 33.9 (*N* = 3)	0.031*
Bite force t^4^ (*N* = 22)	No	63.6 ± 37.5 (*N* = 11)	162.3 ± 119.4 (*N* = 8)	0.007*	66.3 ± 34.1 (*N* = 15)	192.2 ± 63.8 (*N* = 3)	0.002*
Bite force t^5^ (*N* = 23)	No	86.2 ± 48.9 (*N* = 11)	226.6 ± 134.3 (*N* = 9)	0.004*	115 ± 99.9 (*N* = 16)	232.3 ± 74.1 (*N* = 3)	0.047*

Univariate analyses of the qualitative parameters revealed significant associations between normal food intake between four weeks and three months post-surgery and the presence of (partial) osseous non-union (Table 4). Furthermore, recent or former smoking was significantly associated with the presence of (subtotal) osseous non-union between 12 and 24 months post-operatively in the present patient cohort. Post-operative radiotherapy and the chosen plating system showed no significant association with bone healing outcomes.

**Table 4:** Univariate analysis of qualitative parameters grouped after occurrence of incomplete osseous union between 6 and 12 months and 12–24 months with p-values (significant values marked with *) and indication of statistical test

	Categories	Incomplete osseous union 6–12 months (*N* = 24)			Incomplete osseous union 12–24 months (*N* = 23)		
		**No** (*N* = 14)	**Yes** (*N* = 10)	**p-value**	**No** (*N* = 20)	**Yes** (*N* = 3)	**p-value**
Sex	**Male**	6	2	0.388 (Fisher)	7	2	0.538 (Fisher)
	**Female**	8	8		13	1	
History of nicotine abuse	**No**	3	4	0.393 (Fisher)	4	3	0.02* (Fisher)
	**Yes**	11	6		16	0	
History of alcohol abuse	**No**	5	2	0.653 (Fisher)	6	1	1 (Fisher)
	**Yes**	9	8		14	2	
Post-operative radiotherapy	**No**	8	4	0.408 (Chi-squared)	10	1	1 (Fisher)
	**Yes**	6	6		10	2	
Type of plate	**Mix**	11	9	0.615 (Fisher)	15	3	1 (Fisher)
	**Reconstruction plate**	3	1		5	0	
Type of food t^0^	**Normal**	6	5	1 (Fisher)	8	3	0.093 (Fisher)
	**Soft or invasive**	8	5		12	0	
Type of food t^1^	**Normal**	0	1	0.417 (Fisher)	0	0	-
	**Soft or invasive**	14	9		20	3	
Type of food t^2^	**Normal**	0	3	0.059 (Fisher)	0	2	0.012* (Fisher)
	**Soft or invasive**	14	7		20	1	
Type of food t^3^	**Normal**	0	4	0.01* (Fisher)	2	1	0.386 (Fisher)
	**Soft or invasive**	14	4		16	2	
Type of food t^4^	**Normal**	1	4	0.047* (Fisher)	1	3	0.003* (Fisher)
	**Soft or invasive**	12	4		17	0	
Type of food t^5^	**Normal**	1	4	0.116 (Fisher)	3	1	0.47 (Fisher)
	**Soft or invasive**	12	5		16	2	

A multivariate regression using binary logistic analysis was performed to evaluate factors previously identified as significant in univariate analyses or known from the literature as influencing the development of incomplete osseous union. The analysis revealed no significant predictive value for the number of segments (OR 0.461; 95%-CI [0.142; 1.497]; *p* = 0.198), post-operative radiotherapy (OR 0.666; 95%-CI [0.102; 4.340]; *p* = 0.671), type of plate (OR 0.428; 95%-CI [0.032; 5.695]; *p* = 0.521) and history of nicotine abuse (OR 2.368; 95%-CI [0.319; 17,597]; *p* = 0.399) in predicting osseous non-union between six and twelve months (*N* = 24). The ROC-AUC was 0.75 (95%-CI [0.546; 0.954]; *p* = 0.016) indicating an acceptable ability of the regression model to distinguish between osseous non-union and union between six and twelve months. However, the wide 95%-CI in some parameters indicate model instability. Therefore, due to the small number of outcome events, the multivariate model was likely underpowered, and the results of the multivariate analysis should be interpreted with caution.

## Discussion

Mandible reconstruction remains a clinical challenge, particularly due to high rates of osseous non-union [3, 4, 11]. Mechanical signals are known to influence the healing outcome [7–9], and therefore, bite forces are believed to play a role in clinical outcomes. The present study aimed to evaluate bite force and movement parameters over the clinical course and to investigate their relation with bone healing outcomes. The results demonstrated a strong trend in association of incomplete osseous union and excessive bite force.

Previous studies have reported long-term bite force values following mandibular reconstruction [17, 18]. These studies found long-term mean bite force values ranging from 150 N to 190 N. In comparison, the present study found a mean bite force of 154 N in 23 patients after a follow-up period of six months. The difference between 154 N and 190 N is likely explained by the varying follow-up durations of six months to two years [18]. Similar to the present study, Curtis et al. [17] measured the bite force approximately six months after surgery, recording a mean of 150 N. In contrast to previous studies, the present study aimed to quantify the early post-operative bite force as a potential factor influencing the healing outcome. A novel bite force measurement device, previously developed and validated using a universal testing machine with force application up to 600 N, showed deviations between the applied force and the measurement of 0,77% to 5,28%, depending on the applied force and the silicone bite pad thickness [20]. Furthermore, it showed reliable reproducibility results [20]. The device offers the advantage of being applicable in edentulous patients and in those with restricted mouth opening [20]. This makes it particularly suitable for measuring bite force during the initial healing phase after mandibular reconstruction [20]. However, bite force assessment during the early healing phase was only partially feasible, as most patients were initially unable to bite. Two weeks post-surgery, only 9 of 28 patients were able to perform the bite force measurement. At later time points, the number of patients able to perform the biting task increased to 23. Consequently, the mean bite force of 149 N recorded two weeks after surgery does not represent the entire cohort, as 19 of 28 patients were unable to participate at that time. Nevertheless, for the nine patients measured, a significant decrease in bite force (−52%) was observed following surgery, followed by a significant increase (+ 80%) during clinical follow-up. There was a significant trend associating the dental status with pre- and post-operative bite force.

Furthermore, the application of a mean bite force of 150 N two weeks after surgery – corresponding to the average bite force after six months in the entire cohort of the present study and in the study by Curtis et al. [17] – could be suspected to represent an excessive bite force for the initial healing stage. Indeed, in the present study, of the nine patients able to bite two weeks post-operatively, six showed an incomplete osseous union after 6–12 months. These six patients had an average bite force of 166 N at that time. For long bones, interosseous strain favoring bone formation is known to range from 0.03% to 5% [7, 8]. Previous in silico studies reported maximum intersegmental strains of 1.5% during the initial healing phase under titanium patient-specific fixation (the fixators used in the present study) with a bite force of approximately 40–45 N [12, 24]. Assuming a linear relationship between intersegmental strain and bite force, a mean bite force of 166 N would likely approach the upper threshold of acceptable strain (~ 5%). Supplementary finite element analyses of a reconstructed mandible, simulating a reduction in muscle force to 50% of the healthy condition, resulted in a bite force of 177 N and maximum intersegmental strains exceeding 5% (Appendix 1). Thus, intersegmental strains beyond 5% could biomechanically explain the higher rates of incomplete osseous union observed in patients with suspected excessive bite force in the present cohort.

Previous studies investigated the influence of different plating approaches on bone healing outcomes in mandibular reconstruction [10, 12, 24]. However, in the present study, the choice of plating system was not associated with differences in incomplete osseous union. This may be explained by the more substantial effect of excessive bite force - which induces approximately a fourfold increase in intersegmental strain - compared to the variation in plating systems, previously associated with a strain factor increase of around 1.5 [12, 24].

Additionally, statistically significant differences were observed in food intake behavior between patients with complete and incomplete osseous union within the present cohort, particularly during the first three months post-operatively. Consequently, a soft food diet for three months post-operatively could be evaluated in future studies as a clinical strategy to mitigate the risk of mechanical overstimulation. Moreover, the novel bite force measurement device described by Steffen et al. [20] could be utilized pre-operatively to screen patients for high bite force. These patients could benefit from tailored counseling on post-operative behavior to reduce the risk of osseous non-union.

A multivariate analysis of previously described risk factors and those identified as significant in this study - including plating system, post-operative radiotherapy, number of segments, and smoking history - was performed to assess influence factors on the bone healing outcomes between six and twelve months [3, 4, 10, 11, 23, 25]. This analysis did not reveal any significant influence of these factors within the present cohort. Consequently, excessive bite force and food intake behavior appear to be the primary contributors to incomplete osseous union in this patient population. Therefore, limiting excessive bite force post-operatively could be a potential strategy to reduce intersegmental strain and the risk of incomplete osseous non-union. However, wide ranges of 95%-CI in some parameters of the multivariate analysis indicate model instability due to the small number of events. In larger patient cohorts, known influence factors on incomplete osseous union would be suspected to significantly contribute to the ossification outcomes.

The primary limitation of the present prospective study was the absence of functional measurements in many patients, particularly in the early post-operative period. Common reasons for declining the bite force measurement included non-compliance, pain, fear, and restricted mouth opening. Another limitation involved the measurement of functional parameters such as protrusion and laterotrusion, which often yielded small values, making them susceptible to measurement errors. Consequently, these parameters exhibited less consistent trends compared to mouth opening and bite force. Previous studies named different reconstructive techniques and adjuvant radiotherapy as independent risk factors for the development of incomplete osseous union [23, 26]. Within the multivariate analysis, due to the small number of events of this secondary end point, these risk factors were not significantly associated with incomplete osseous union. Future studies should evaluate incomplete osseous union as primary end point using a more sophisticated study design. Using this approach, the known risk factors would likely be significantly associated with incomplete osseous union. However, in future studies investigating bite force measurements over time, ethical considerations need to be taken into account, since patients should not feel encouraged to permanently and unphysiologically load the healing mandible. Although primary mandibular reconstruction has been evaluated within the present study, secondary mandibular reconstruction using a temporary reconstruction plate represents a clinical alternative [27]. However, this approach is not free of complications, for example the risk of cutaneous perforation is significantly increased in comparison to primary reconstructive strategies including osseous flaps fixed with plates [27]. Furthermore, incomplete osseous union has been investigated as a secondary end point along with the functional measurements in the clinical course after mandibular reconstruction. Therefore, patients with primarily reconstructed mandibles using osseous flaps have been chosen as subjects within the present study.

In conclusion, mandibular reconstructive surgery resulted in a significant decrease in all investigated functional parameters - bite force, mouth opening, protrusion, and laterotrusion. Within the first six months post-operatively, these functional parameters showed significant recovery. A significant trend in association of dental status and bite force was observed. Bite forces > 150 N during the clinical course were in the present cohort observed to be associated with incomplete osseous union. A potential explanation could be excessive intersegmental strain resulting in a lack of bone formation. Pre-operative screening for high bite force, combined with a recommendation for a soft food diet during the first three months after mandibular reconstruction has been identified as a potential strategy to permanently reduce possibly excessive mechanical stimuli.

## Figures and Tables

**Fig. 5:**
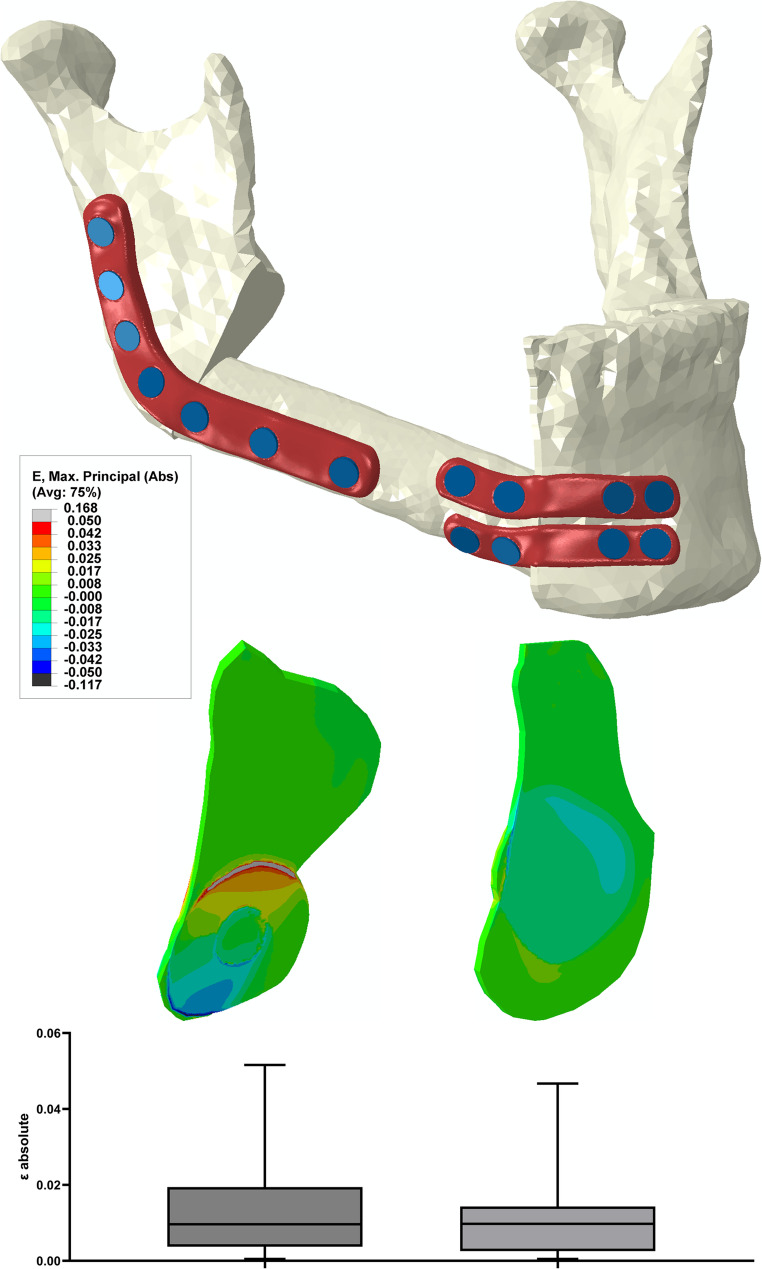
Strain distributions in anterior (right) and posterior (left) intersegmental gaps in a one-segmental mandibular reconstruction under fixation with a combination of a short posterior titanium reconstruction plate with anterior titanium miniplates and a bite force of 177 N

## Data Availability

Data is provided within the manuscript. Raw data cannot be shared to protect study participant privacy as direct patient data was involved in the analysis.

## Appendix

Previous studies have investigated mandibular reconstruction in a single-segment scenario in the initial healing phase, assuming a muscle force reduction from a healthy state to 12.5% [12]. This resulted in a bite force of approximately 45 N, which was considered a valid value for the initial healing phase compared to the long-term bite force values of 150–190 N reported after mandibular reconstruction [17, 18]. Furthermore, in cases of fracture repositioning, early post-operative measurements (two weeks) reported bite forces of approximately 40 N [28].

Since some patients in the present study exhibited excessively high bite force values during the initial healing phase and showed increased rates of incomplete osseous union, additional computational analyses were performed, assuming a muscle force reduction to 50% instead of 12.5% during this phase [12]. This resulted in a bite force of 177 N, with observed intersegmental strain values reaching up to 5% (Appendix 1). For long bones, detailed strain thresholds associated with varying cell differentiation patterns have been described [8]. Strain values exceeding 5% have been linked to fibrous tissue formation, which could explain the incomplete osseous union rates observed in the present study, predominantly in the group with excessive post-operative bite force.

## References

[1] Brown JS, Lowe D, Kanatas A, Schache A (2017) Mandibular reconstruction with vascularised bone flaps: a systematic review over 25 years. Br J Oral Maxillofac Surg 55:113–126 28065645 10.1016/j.bjoms.2016.12.010

[2] Rendenbach C, Hölterhoff N, Hischke S, Kreutzer K, Smeets R, Assaf AT, Heiland M, Wikner J (2018) Free flap surgery in europe: an interdisciplinary survey. Int J Oral Maxillofac Surg 47:676–682 29275838 10.1016/j.ijom.2017.11.013

[3] Knitschke M, Sonnabend S, Roller FC, Pons-Kühnemann J, Schmermund D, Attia S, Streckbein P, Howaldt H-P, Böttger S (2022) Osseous union after mandible reconstruction with fibula free flap using manually bent plates vs. patient-specific implants: a retrospective analysis of 89 patients. Curr Oncol (Toronto Ont) 29:3375–3392 10.3390/curroncol29050274 PMC9139377 35621664

[4] Knitschke M, Yonan M, Roller FC, Pons-Kühnemann J, Attia S, Howaldt H-P, Streckbein P, Böttger S (2022) Osseous union after jaw reconstruction with Fibula-Free flap: conventional vs. CAD/CAM Patient-Specific implants. Cancers 14 10.3390/cancers14235774 PMC9738514 36497255

[5] Kreutzer K, Steffen C, Nahles S, Koerdt S, Heiland M, Rendenbach C, Beck-Broichsitter B (2022) Removal of patient-specific reconstruction plates after mandible reconstruction with a fibula free flap: is the plate the problem? Int J Oral Maxillofac Surg 51:182–190 33933334 10.1016/j.ijom.2021.04.003

[6] Claes L (2017) [Mechanobiology of fracture healing part 2 : Relevance for internal fixation of fractures]. Unfallchirurg 120:23–31 27975121 10.1007/s00113-016-0281-2

[7] Claes L (2017) [Mechanobiology of fracture healing part 1 : Principles]. Unfallchirurg 120:14–22 27966008 10.1007/s00113-016-0280-3

[8] Claes LE, Heigele CA (1999) Magnitudes of local stress and strain along bony surfaces predict the course and type of fracture healing. J Biomech 32:255–266 10093025 10.1016/s0021-9290(98)00153-5

[9] Duda GN, Geissler S, Checa S, Tsitsilonis S, Petersen A (2023) and K. Schmidt-Bleek, the decisive early phase of bone regeneration. Nat Rev Rheumatol 10.1038/s41584-022-00887-0 36624263

[10] Steffen C, Fischer H, Sauerbrey M, Heintzelmann T, Voss JO, Koerdt S, Checa S, Kreutzer K, Heiland M, Rendenbach C (2022) Increased rate of pseudarthrosis in the anterior intersegmental gap after mandibular reconstruction with fibula free flaps: a volumetric analysis. Dento Maxillo Fac Radiol 51:20220131 10.1259/dmfr.20220131 PMC9522980 35762353

[11] Rendenbach C, Steffen C, Hanken H, Schluermann K, Henningsen A, Beck-Broichsitter B, Kreutzer K, Heiland M, Precht C (2019) Complication rates and clinical outcomes of osseous free flaps: a retrospective comparison of CAD/CAM versus conventional fixation in 128 patients. Int J Oral Maxillofac Surg 48:1156–1162 30792087 10.1016/j.ijom.2019.01.029

[12] Ruf P, Orassi V, Fischer H, Steffen C, Duda GN, Heiland M, Kreutzer K, Checa S, Rendenbach C (2022) Towards mechanobiologically optimized mandible reconstruction: CAD/CAM miniplates vs. reconstruction plates for fibula free flap fixation: A finite element study. Front Bioeng Biotechnol 10 10.3389/fbioe.2022.1005022 PMC9712730 36466355

[13] Kennady MC, Tucker MR, Lester GE, Buckley MJ (1989) Stress shielding effect of rigid internal fixation plates on mandibular bone grafts. A photon absorption densitometry and quantitative computerized tomographic evaluation. Int J Oral Maxillofac Surg 18:307–310 2509587 10.1016/s0901-5027(89)80101-8

[14] Schupp W, Arzdorf M, Linke B, Gutwald R (2007) Biomechanical testing of different osteosynthesis systems for segmental resection of the mandible. J Oral Maxillofac Surg 65:924–930 17448842 10.1016/j.joms.2006.06.306

[15] Yoda N, Zheng K, Chen J, Liao Z, Koyama S, Peck C, Swain M, Sasaki K, Li Q (2018) Biomechanical analysis of bone remodeling following mandibular reconstruction using fibula free flap. Med Eng Phys 56:1–8 29609866 10.1016/j.medengphy.2018.03.008

[16] Zheng K, Yoda N, Chen J, Liao Z, Zhong J, Wu C, Wan B, Koyama S, Sasaki K, Peck C, Swain M, Li Q (2022) Bone remodeling following mandibular reconstruction using fibula free flap. J Biomech 133:110968 35139441 10.1016/j.jbiomech.2022.110968

[17] Curtis DA, Plesh O, Miller AJ, Curtis TA, Sharma A, Schweitzer R, Hilsinger RL, Schour L, Singer M (1997) A comparison of masticatory function in patients with or without reconstruction of the mandible. Head Neck 19:287–296 9213107 10.1002/(sici)1097-0347(199707)19:4<287::aid-hed7>3.0.co;2-x

[18] Sakuraba M, Miyamoto S, Fujiki M, Higashino T, Oshima A, Hayashi R (2017) Analysis of functional outcomes in patients with mandible reconstruction using vascularized fibular grafts. Microsurgery 37:101–104 26052686 10.1002/micr.22433

[19] Aftabi H, Zaraska K, Eghbal A, McGregor S, Prisman E, Hodgson A, Fels S (2024) Computational models and their applications in biomechanical analysis of mandibular reconstruction surgery. Comput Biol Med 169:107887 38160502 10.1016/j.compbiomed.2023.107887

[20] Steffen C, Duda K, Wulsten D, Voss JO, Koerdt S, Nahles S, Heiland M, Checa S, Rendenbach C (2023) Clinical and technical validation of novel bite force measuring device for functional analysis after mandibular reconstruction. Diagnostics (Basel Switzerland) 13 10.3390/diagnostics13040586 PMC9955263 36832074

[21] Lampert P, Fenske J, Wuster J, Koerdt S, Kreutzer K, Ruf P, Checa S, Heiland M, Steffen C, Rendenbach C (2024) Comparative study of CAD/CAM reconstruction and miniplates for patient-specific fixation in LCL-type mandibular reconstruction. Front Oncol 14:1438269 39323993 10.3389/fonc.2024.1438269 PMC11422126

[22] Boyd JB, Gullane PJ, Rotstein LE, Brown DH, Irish JC (1993) Classification of mandibular defects. Plast Reconstr Surg 92:1266–1275 8248401

[23] Kreutzer K, Lampert P, Doll C, Voss JO, Koerdt S, Heiland M, Steffen C, Rendenbach C (2023) Patient-specific 3D-printed mini-versus reconstruction plates for free flap fixation at the mandible: retrospective study of clinical outcomes and complication rates. J Craniomaxillofac Surg 51:621–628 37852889 10.1016/j.jcms.2023.09.019

[24] Ruf P, Orassi V, Fischer H, Steffen C, Kreutzer K, Duda GN, Heiland M, Checa S, Rendenbach C (2024) Biomechanical evaluation of CAD/CAM magnesium miniplates as a fixation strategy for the treatment of segmental mandibular reconstruction with a fibula free flap. Comput Biol Med 168:107817 38064852 10.1016/j.compbiomed.2023.107817

[25] Fenske J, Steffen C, Mrosk F, Lampert P, Nikolaidou E, Beck M, Heiland M, Kreutzer K, Doll C, Koerdt S, Rendenbach C (2025) A critical reflection of radiotherapy on osseous free flaps in mandibular segmental resection and immediate reconstruction in locally advanced oral squamous cell carcinoma: a cohort study. Radiother Oncol 202:110652 39586357 10.1016/j.radonc.2024.110652

[26] Fenske J, Lampert P, Nikolaidou E, Steffen C, Beck M, Neckel N, Nahles S, Heiland M, Mrosk F, Koerdt S, Rendenbach C (2025) Osteoradionecrosis in osseous free flaps after maxillofacial reconstruction: a single-center experience. Front Oncol 15:1527149 39949741 10.3389/fonc.2025.1527149 PMC11821973

[27] Ritschl LM, Mucke T, Hart D, Unterhuber T, Kehl V, Wolff KD, Fichter AM (2021) Retrospective analysis of complications in 190 mandibular resections and simultaneous reconstructions with free fibula flap, Iliac crest flap or reconstruction plate: a comparative single centre study. Clin Oral Investig 25:2905–2914 33025147 10.1007/s00784-020-03607-8 PMC8060197

[28] Gheibollahi H, Aliabadi E, Khaghaninejad MS, Mousavi S, Babaei A (2021) Evaluation of bite force recovery in patients with maxillofacial fracture. Journal of cranio-maxillo-facial surgery. official publication of the European Association for Cranio-Maxillo-Facial Surgery 10.1016/j.jcms.2021.02.017 33653602

